# Response of the Bacterial Community and Antibiotic Resistance in Overnight Stagnant Water from a Municipal Pipeline

**DOI:** 10.3390/ijerph17061995

**Published:** 2020-03-18

**Authors:** Minglu Zhang, Mengyao Xu, Shaofeng Xu, Lingyue Zhang, Kaizong Lin, Lei Zhang, Miao Bai, Can Zhang, He Zhou

**Affiliations:** 1 Department of Environmental Science and Engineering, Beijing Technology and Business University, Beijing 100048, China; zhangminglu@th.btbu.edu.cn (M.Z.); x13701140180@163.com (M.X.); xushaofeng9611@126.com (S.X.); zly19800371007@163.com (L.Z.); 17854117307@163.com (K.L.); 2 Center for Disease Control and Prevention of Chinese PLA, Beijing 100071, China; zhanglei332700@163.com (L.Z.); baimiaobtbu@163.com (M.B.); 3 Beijing Boda Water Company, Beijing 100176, China; zhouhe@bdawater.com

**Keywords:** drinking water, overnight stagnation, pipeline water, heterotrophic plate count (HPC), antibiotics, bacterial community composition

## Abstract

Although drinking water safety has raised considerable concern, to date, the hidden health risks in newly released overnight water from a municipal pipeline have seldom received attention. In this study, bacterial community composition and the response of antibiotic-resistant bacteria (ARB) to ciprofloxacin, azithromycin, tetracycline, penicillin, and cephalosporin in overnight stagnant water were analyzed. With increases in heterotrophic bacteria plate count (HPC) during water stagnation, the numbers of ARB and the ARB/HPC ratios for the five antibiotics in resident water were observed to increase, which illustrated that the prevalence of ARB rose in the pipe network water during stagnation time (ST). Furthermore, during water stagnation for 12 h, an increase in bacteria related to fermentation was also observed. When the ST rose to 48 h, the fermentation bacteria become non-significant, and this was related to the exchange of pipe network water during daytime stagnation within the 48-h period. The antibiotic resistance index (ARI) showed that tetracycline had the highest resistance level in fresh water, and then decreased during water stagnation. When ST increased to 12 h, all ARI values of the five antibiotics were low, which was associated with changes in parameters during water retention and reduced resistance during short-term stagnation. When the ST increased to 24 and 48 h, the resistance to most antibiotics (except for tetracycline) increased, which showed that increasing antibiotic resistance is caused by the formation of biofilms in the pipeline during water stagnation.

## 1. Introduction

Drinking water safety problems have a considerable impact on public health. However, the safety of overnight stationary water in municipal pipelines is a relatively neglected issue. For most people, the first thing they do after getting up in the morning is to turn on the faucet, wash their face, brush their teeth, boil water and even drink tap water directly. These same procedures are performed when people return to their house or office buildings without considering the safety of tap water which has been in the pipe network for days. Little is known about the hidden health risks of newly released overnight water from municipal pipelines, and the deterioration of resident water quality has also been neglected. In previous research, heavy metal concentrations in household stationary tap water were investigated, and increased contamination by metals such as lead, cadmium, copper and nickel was detected in pipeline water after overnight stagnation [1,2,3]. 

However, contamination by microbes in resident water should raise more concern. Compared with flowing fresh water, still resident water in pipelines contributes to the rapid proliferation of microorganisms. During stagnation, the pipe network acts like a giant stationary reactor, providing a continuous and stable environment for microorganism growth. This contributes not only to the augmentation of planktonic bacteria, but also to the growth of biofilms on the wall of the pipeline [4]. Furthermore, when the faucet is turned on after several days, biofilms which are not firmly attached will detach and fall into the flowing water and increase the health risks associated with microbes. It was reported that biofilms derived from the pipe network not only result in turbid water containing pathogenic bacteria, which pose a serious threat to human health, but also antibiotic-resistant bacteria (ARB) such as *Escherichia coli, Acinetobacter*, *Pseudomonas*, and *Sphingomonadaceae* [5,6,7]. The proliferation of ARB in the drinking water distribution system triggers the spread of antibiotic resistance genes (ARGs). ARGs can be persistent in drinking water, combined with movable gene elements such as plasmids, transposons, integrons, etc., and can migrate from one species to another or even among genus [8,9]. A variety of ARGs have been detected in drinking water systems, including tetracycline, aminoglycoside, chloramphenicol, vancomycin, thiamine, and penicillin resistance genes [10,11]. Xi et al. reported that the relative abundance of ARGs was found in water from the pipe network were higher than in wastewater treatment plant effluents and water sources, which was mainly due to biofilm growth in the pipeline system [6]. Zhang et al. reported that during 120 days of continuous operation of a simulated drinking water distribution system, six ARGs (*ermA*, *ermB*, *aphA2*, *ampC*, *sulII*, and *tetO*) in samples of three phases (water, particle, and biofilm) from the reactor increased [12]. However, to date, the differences in the bacterial community composition and antibiotic resistance between fresh water and resident water are unclear. In this study, overnight water with different stagnation times (STs) was sampled from Beijing, China, and bacterial community composition and antibiotic resistance in ARB cultivable microorganisms were analyzed, with the aim of determining the risk of bacterial proliferation and antibiotic resistance in response to overnight stagnant water from a municipal pipeline.

## 2. Materials and Methods

### 2.1. Sample Collection

Samples of fresh water and resident water were collected from a faucet in a laboratory in the Haidian district of Beijing, China, which had a direct water supply from the municipal pipe network. The main quality indices for fresh water were as follows: turbidity: 0.35–0.37 NTU (nephelometric turbidity units); pH value: 7.91–8.79; total dissolved solids: 331–403 mg/L; sulfate: 47–66 mg/L; chloride: 39–51 mg/L; fluoride: 0.26–0.49 mg/L; nitrate nitrogen: 5.13–7.50 mg/L; iron: 0–0.05 mg/L; manganese: 0.004–0.005 mg/L; copper: 0.034–0.053 mg/L; zinc: 0.17–0.99 mg/L.

Fresh water W_0_ represents water standing for 0 h ST, and resident water with 12 h ST, 24 h ST and 48 h ST, were represented by W_ST-12h_, W_ST-24h_ and W_ST-48h_, respectively. W_0_ is representative of fresh water and was collected at 9:00 pm of the first day. The collection method used for W_0_ was according to the standard examination methods for drinking water [13]. This method was as follows: after turning on the faucet for 10 min, 10 to 40 Liters of fresh water was collected in a disposable sterilized water bag. The resident water W_ST-12h_ was collected at 9:00 am on the second day, and this water had been standing overnight with a 12 h ST, namely one night. The resident water W_ST-24h_ was representative of overnight water with a 24 h ST, namely one day and one night, and was collected at 9:00 am on the third day. The resident water W_ST-48h_ was collected at 9:00 am on the fifth day, and was representative of overnight water with a 48 h ST, namely two days and two nights. The collection method used for W_ST-12h_, W_ST-24h_ and W_ST-48h_ was as follows: after 10 liters of resident water was collected in a disposable sterilized water bag, the faucet was turned on for 10 min with the aim of reducing the interference of resident water in the next sample collection. All samples of W_0_, W_ST-12h_, W_ST-24h_ and W_ST-48h_ were collected four times, and the average values were calculated.

### 2.2. Turbidity and Residual Chlorine 

Turbidity and residual chlorine of the water samples were measured with a HACH 1900C (Hach, New York, USA) portable turbidity meter and a HACH 5870000 (Hach, New York, USA) portable residual chlorine analyzer, respectively (GB/T5750-2006). 

### 2.3. Heterotrophic Bacteria Plate Count (HPC)

The water samples were each filtered using a triple stainless-steel filter and a polyether sulfone membrane with a pore size of 0.22 μm. Water (100 mL) was passed through each filter. The filter was then placed on a solid medium and cultured at 22 °C for 5 days. HPC was reported as colony-forming units (CFU)/mL.

### 2.4. DNA Extraction and High-Throughput Sequencing

The collected 10 to 40 L drinking water samples were filtered through a 0.22-μm pore size membrane (47 mm diameter, Millipore, Burlington, MA, USA) and a 90-mm filter holder (Millipore, USA) to concentrate the bacterial cells, which were then mixed with purified water (5 mL). The filtered membrane was ultrasonically shaken for 20 min (40 kHz) to separate bacterial cells attached to the membrane. The enriched samples were then subjected to total DNA extraction using the Fast DNA SPIN kit (MP Bio, Irvine, CA, USA) according to the manufacturer’s instructions. The purified DNA concentration was measured by NanoDrop 2000 spectrophotometry (Thermo Fisher Scientific, Wilmington, DE, USA), and the DNA was stored at −20 °C until subsequent analysis.

Each DNA sample was amplified using the HotStarTaq Plus Master Mix Kit (Qiagen, USA), the 515F/806R primer set with a unique barcode were used to amplify a variable region of the 16SrRNA genes. PCR amplification conditions were: 1 cycle of 94 °C for 10 min, followed by 25 cycles of 94 °C for 30 s, 55 °C for 15 s and 72 °C for 30 s. Amplicons were pooled in equal proportions and purified using Ampure XP beads (Agencourt). The purified product was used to prepare the Illumina DNA library. Libraries were sequenced on the Illumina Hiseq 2500 platform (Novogene, Beijing, China) using a read length up to 2 × 250 bp. Sequencing data processing was performed with QIIME by using the default parameters to trim off the low quality reads, adaptors, barcodes and primers. The obtained high-quality effective sequences were classified into the same operational taxonomic units (OTUs) at an identity threshold of 97% similarity. The sequence with the highest abundance in each OTU was picked out and regarded as the representative sequence. The estimated richness by Mothur was used for comparisons among samples. The taxa-relative abundances were generated into phylum, class, order, family, and genus levels for each sample. Alpha diversity was described for each sample using metrics include OTUs, Chao1, Shannon index and PD Whole tree, and rarefaction curves were generated to compare the level of bacterial OTU diversity.

### 2.5. ARB Calculation of the Antibiotic Resistance Rate

#### 2.5.1. Antibiotic Exposure 

Five commonly used antibiotics in the treatment of human disease were selected, including ciprofloxacin, azithromycin, tetracycline, penicillin and cephalosporin. The antibiotic concentrations for ARB enumeration were determined by the Clinical and Laboratory Standards Institute (CLSI) [14]. The concentration of each antibiotic was tested in triplicate and the results averaged. The initial concentration of the five antibiotics was 500 mg/L. Ciprofloxacin was treated with methanol as a solvent to ensure that its concentration in the culture dish was 4 mg/L. The solvent used for both azithromycin and tetracycline was 70% ethanol, and the concentrations in the culture dish were 8 and 16 mg/L, respectively. The solvent for penicillin and cephalosporin was 100% water, and the concentrations in the culture dish were 16 and 32 mg/L, respectively.

#### 2.5.2. Quantitative Assessment of Antibiotic Resistance

For HPC in W_0_, W_ST-12h_, W_ST-24h_ and W_ST-48h_, their antibiotic exposure under a series of concentrations (0, 4, 8, 16 and 32 mg/L) was determined. After incubation with five antibiotics at 22 °C for 24 h, the survival rate (SR) of HPC in different samples was calculated by the formula, shown by Equation (1).

(1) $$\mathrm{SR}\;\left(\%\right)=\frac{N_{i}}{N_{0}}\times 100\%\;\;\;\;\;\;\;\;\;$$

where *N*_i_ is the HPC response to five antibiotics, including ciprofloxacin, azithromycin, tetracycline, penicillin, and cephalosporin. *N*_0_ is the HPC response without antibiotics.

The degree of HPC response to different antibiotic doses was mathematically fitted through a four-parameter logistic function as shown in Equation (2) [15].

(2) $$\mathrm{SR}\;\left(\%\right)=\frac{A_{1}-A_{2}}{1+{\left(X/IC_{50}\right)}^{p}}+A_{2}$$

SR was acquired using Equation (1); A_1_ represents an antibiotic-free reaction with an initial value of 100; A_2_ represents “infinite dose” with an initial value of 0; IC_50_ represents 50% inhibitory concentration; X represents an arithmetic dose; and p represents the “Slope factor”.

The exposure of HPC in water samples at different antibiotic concentrations was fitted by Equation (2) to obtain the IC_50_ of HPC to an antibiotic, and this was used as the composite antibiotic tolerance of HPC. Logistic dose-responses to antibiotics were fitted by Origin 8.0 (Hampton, MA, USA).

When the relative antibiotic resistance of HPC was compared to the antibiotic resistance standard provided by CLSI, the antibiotic resistance index (ARI) can be used for evaluation. The definition of ARI is shown in Equation (3).

(3) $$ARI=\frac{IC_{50}}{MIC\;}$$

Here, MIC represents the minimum inhibitory concentration, and the maximum MIC of a typical pathogen was determined from the CLSI report. The MIC of ciprofloxacin, azithromycin, tetracycline, penicillin, and cephalosporin were 4, 8, 16, 16, and 32 mg/L, respectively.

### 2.6. Statistical Analysis 

Microsoft Excel 2010 was used for data analysis. Canoco version 5.0 software was used for redundancy analysis (RDA) to check the correlation between bacterial communities and antibiotic resistance.

## 3. Results and Discussion

### 3.1. Turbidity, HPC and Residual Chlorine 

**Figure 1:**
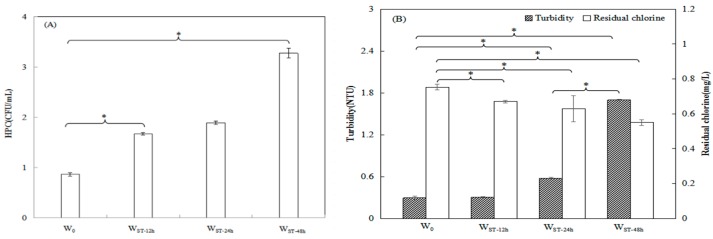
Heterotrophic bacteria plate count (HPC), turbidity and residual chlorine in samples of W_0_, W_ST-12h_, W_ST-24h_ and W_ST-48h_ with different STs. (**A**) HPC. (**B**) Turbidity and residual chlorine. The values of the bars are the average measurements and error bars are standard deviations of 4 samples collected four times. *Represents P < 0.05.

As shown in Figure 1, a gradual increasing trend in HPC and turbidity in the water samples was observed with increased ST. HPC in W_ST-48h_ increased markedly to 3.3 CFU/mL compared with 0.9 CFU/mL of HPC in W_0_ (P < 0.05) (Figure 1A). As reported by Karin et al., HPC in tap water sampled from 10 separate households were below the limit of 9.1–9.8 CFU/mL, and an obvious increase (4–580-fold) was detected in the stagnant water samples, which suggests microbial growth during overnight stagnation [16]. The HPC in W_ST-12h_ increased compared with that in W_0_, and the difference was statistically significant (P < 0.05). This demonstrated that microbes significantly increased after 12 h of overnight stagnation (Figure 1A). However, this value was not significantly different compared with that in W_ST-24h_ (P > 0.05). This showed that the HPC did not increase significantly from 12 h to 24 h stagnation, as the pipeline water retained in daytime is not under the same conditions as that retained overnight. During 24 h stagnation, the portion of pipeline water retained during daytime is influenced by nearby faucets which are used under normal service conditions. Another part of resident water far from the turned-off faucets is in an intermediate state between still and flowing. Only the water near the turned-off faucets is still in the pipeline during the daytime in the 24 h stagnation period. However, this intricate state of W_ST-24h_ was not observed in the overnight stagnation of W_ST-12h_, as almost all of the faucets near the sampling faucets were turned off at nighttime. 

Turbidity in W_ST-48h_ reached 1.7 NTU compared with 0.3 NTU in W_0_, and this difference was statistically significant (P < 0.05) (Figure 1B). Turbidity in W_ST-12h_ increased compared with that in W_0_ and was not statistically significant (P > 0.05). However, turbidity in W_ST-24h_ and W_ST-48h_ increased compared with that in W_0_ and these increases were statistically significant (P < 0.05) (Figure 1B). This suggested that the detachment of biofilms was not detected during 12 h stagnation, and may have occurred at 24 h and 48 h stagnation. The proliferation of microorganisms occurred in the pipeline water during overnight stagnation, and the resident water was suitable for bacterial growth and reproduction. Either planktonic growing bacteria in the pipeline water or microbial growth and subsequent detachment in the biofilms on the pipes triggered the increase in HPC and turbidity during stagnation.

Furthermore, the residual chlorine in W_0_, W_ST-12h_, W_ST-24h_ and W_ST-48h_ gradually declined with increasing ST. It dropped from 0.8 mg/L in W_0_ to 0.6 mg/L in W_ST-48h_ and this decrease was statistically significant (P < 0.05) (Figure 1B). This indicated that increasing turbidity and HPC during the different STs leads to more chlorine consumption when residual water is still in the pipe network [12]. The residual chlorine in W_ST-12h_ decreased compared with that in W_0_ and the difference was statistically significant (P < 0.05), which demonstrated that microbes significantly increase after overnight stagnation for one night (Figure 1B). With regard to W_ST-24h_, residual chlorine decreased significantly compared with W_0_ (P < 0.05), while compared with W_ST-12h_, no significant difference was observed (P > 0.05). The cause of this was similar to that for HPC variations in W_ST-12h_ and W_ST-24h_. 

### 3.2. Response of the Bacterial Community 

#### 3.2.1. Bacterial Phyla

**Figure 2:**
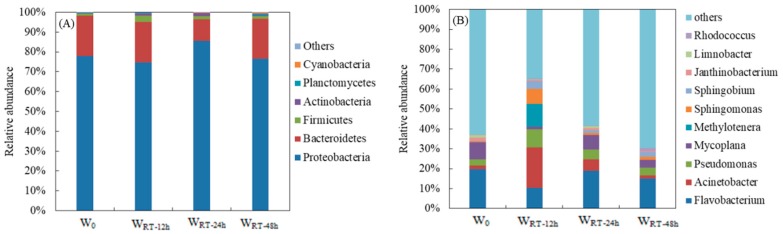
Bacterial community composition in W_0_, W_ST-12h_, W_ST-24h_ and W_ST-48h_ water samples with different STs. (**A**) Relative abundance of different phyla. Sequences not classified to any known phylum and rare species with relative abundance less than 0.1% are included as others. (**B**) Relative abundance of different genera. Sequences not classified to any known genus and rare species with relative abundance less than 0.5% are included as others.

As shown in Figure 2A, a total of six bacterial phyla (average relative abundance > 0.1%) were frequently identified in W_0_, W_ST-12h_, W_ST-24h_ and W_ST-48h_, including *Proteobacteria* (74.68%–85.57%), *Bacteroidetes* (10.91%–20.46%), *Firmicutes* (0.99%–3.26%), *Actinobacteria* (0.39%–1.48%), *Planctomycetes* (0.08%–0.69%) and *Cyanobacteria* (0.08%–0.45%). The predominant bacteria in these water samples were *Proteobacteria* and *Bacteroidetes* [17]. In various publications, *Proteobacteria* were the most predominant taxa in drinking water bacterial communities [18], and members of the phylum *Bacteroidetes* were also among the predominant bacteria in drinking water [19,20]. In addition, *Firmicutes* was a major group in the biofilm of drinking water distribution system pipes transporting treated surface waters, and was a minor bacterial group in the biofilm of drinking water distribution system pipes transporting treated ground waters [21]. Other phyla with low relative abundance, such as *Actinobacteria*, *Planctomycetes* and *Cyanobacteria*, have been reported in drinking water [21,22,23]. For example, Kahlisch et al. reported that the bacterial community in tap water was dominated by *Proteobacteria*, *Cyanobacteria* and *Bacteroidetes*, followed by *Chloroflexi*, *Nitrospira*, *Firmicutes* and *Planctomycetes*. *Betaproteobacteria* in *Proteobacteria* formed the largest fraction, with an average of 21% of the total community, followed by *Cyanobacteria* (16%), *Alphaproteobacteria* (15%), *Gammaproteobacteria* (10%), and *Bacteroidetes* (8%) [17].

The ST had an effect on the abundance of dominant bacterial phyla (Figure 2A). The variations in *Bacteroidetes* and *Firmicutes* in W_ST-12h_ and W_ST-24h_ are concerning. As shown in previous investigations, *Firmicutes* was negatively correlated with alkalinity and positively correlated with organic matter [21], and had a significant positive correlation with nitrite nitrogen [22]. It was also reported that both *Bacteroides* and *Firmicutes* are important in the fermentation reaction, e.g., the relative abundance of *Bacteroides* and *Firmicutes* in intestinal flora are closely related to fat deposition, and the ratio of the two (*Bacteroidetes*/*Firmicutes*) may be even more important [24]. Zlatanović research found that the total organic carbon (TOC) concentrations decreased with the stagnation time of two taps, as a commonly applied measure to indicate the natural organic matter (NOM) concentration in a water sample [4]. Meanwhile, Prest suggested that 1 μg/L of organic carbon is enough to stimulate the growth of 10^3^–10^4^ cell/mL [25]. The TOC of tap water in this study has been revealed in previous experiments to be 1.1 mg/L [12]. The highest relative abundance of *Firmicutes* occurred in W_ST-12h,_ and then decreased in W_ST-24h_, which suggests an increase in organic matter in W_RT-12_ due to 12 h stagnation over one night, which was then reduced at 24 h stagnation due to flowing water during the daytime. With regard to the ratio of *Bacteroidetes*/*Firmicutes*, the value of 20.5 in W_0_ gradually decreased to 6.3 in W_ST-12h_, and then increased to 7 in W_ST-24h_ and 16 in W_ST-48h_ (data not shown), which showed that the lowest ratio of *Bacteroidetes*/*Firmicutes* was detected in W_ST-12h_, followed by W_ST-24h_. These results suggest that an increase in fermentation reactions occurred in W_ST-12h_, mainly due to overnight stagnation. However, when the ST increased to 48 h, the repeat exchange of flowing water during the two daytime periods resulted in the fermentation reactions in W_ST-48h_ being non-significant.

#### 3.2.2. Bacterial Genera

Figure 2B illustrates the relative abundance of different genera in W_0_, W_ST-12h_, W_ST-24h_ and W_ST-48h_. The dominant bacteria (average relative abundance > 0.5%) contained 10 genera, including *Flavobacterium* (10.44%–19.72%), *Acinetobacter* (1.51%–20.28%), *Pseudomonas* (3.32%–8.94%), *Mycoplana* (1.32%–8.45%), *Methylotenera* (0.10%–11.58%), and *Sphingomonas* (0.50%–7.49%). As previous reports have shown, species such as *Flavobacterium*, *Acinetobacter*, *Pseudomonas*, *Methylotenera* and *Sphingomonas* were frequently detected, and were common species in tap water [26,27]. For example, an investigation of the bacterial community in nine drinking water wells in Mexico showed that *Pseudomonas* was the dominant genus, and *Flavobacterium* was a genus with high abundance in tap water [28]. 

The ST has an effect on the dominant bacterial genera (Figure 2B). Compared with W_0_, when the ST increased to 12 h, the relative abundance of four genera (*Acinetobacter*, *Methylotenera, Pseudomonas* and *Sphingomonas*) increased, and of these genera, the abundance of *Methylotenera* and *Sphingomonas* significantly increased by up to 110 times and 15 times, respectively (P < 0.05). When ST increased to 48 h, three dominant genera (*Acinetobacter*, *Pseudomonas*, and *Sphingomonas*) decreased compared to that in W_ST-24h_, which was related to daytime stagnation due to the flow and consumption of water during the daytime. In contrast, compared with W_0_, when the ST increased to 12 h, the relative abundance of *Flavobacterium* and *Mycoplana* reduced, and then increased at 24 h, which showed that *Flavobacterium* and *Mycoplana* may not be suited to nighttime stagnation. *Pseudomonas* was the most abundant genus on the biofilm cultured in the reactor of a laboratory simulated drinking water distribution system [29,30]. Zhang et al. found a high level of *Sphingomonas* in biofilms on the walls of a water supply clear well in a drinking water treatment plant with chlorine disinfection [27]. As shown by a previous investigation, chlorination increased the relative abundance of *Pseudomonas* and *Sphingomonas* in drinking water [18]. In addition, Chao et al. found that *Sphingomonas* was one of the most abundant genera in biofilms and stated that *Sphingomonas* was dominant only at the early stage of the biofilm, while the relative abundance decreased significantly in older biofilms [29]. As shown in Figure 2B, *Pseudomonas* and *Sphingomonas* in W_ST-12h_ were higher than those in W_0_, which may be due to biofilm growth in the pipeline during water stagnation. In addition, *Sphingomonas* abundance in W_ST-12h_ was higher than that in W_ST-24h_ and W_ST-48h_ (Figure 2), which is in agreement with the report by Chao et al [29]. *Methylotenera* have also been reported to contribute to organic matter biodegradation due to methylotrophic groups that are obligate methyl utilizers [31,32]. High abundance of *Methylotenera* was observed in W_ST-12h,_ which demonstrated that probably more organic matter degradation occurred during 12 h overnight stagnation.

### 3.3. Antibiotic Resistance 

#### 3.3.1. ARB Distribution 

**Figure 3:**
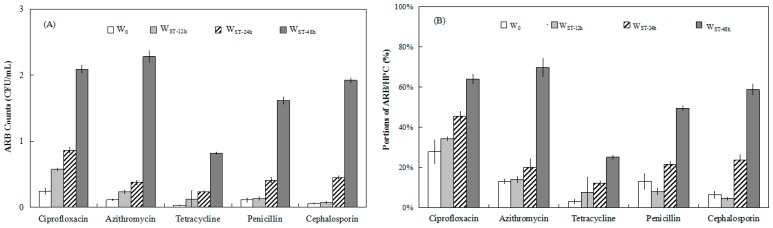
Antibiotic-resistant bacteria (ARB) distribution in water samples with different STs. (**A**) Counts of ARB. (**B**) ARB/HPC ratios. The values of the bars are the average measurements and error bars are standard deviations of 4 samples collected four times.

The counts of ARB resistant to ciprofloxacin, azithromycin, tetracycline, penicillin, and cephalosporin were 0.03–0.2 CFU/mL in W_0_, while 0.1–0.6, 0.2–0.9, and 0.8–2.3 CFU/mL of ARB counts were detected in W_ST-12h_, W_ST-24h_ and W_ST-48h_, respectively, which was higher than the ARB counts in W_0_ (Figure 3A). Their ratios of ARB to HPC (ARB/HPC) were 3.2%–27.6% in W_0_, while the detection rate for W_ST-12h_, W_ST-24h_ and W_ST-48h_ were 7.5%–34.4%, 12.3%–45.6% and 25.1%–69.8%, respectively, which were higher than the ARB/HPC ratio in W_0_ (Figure 3B). The enhanced ARB amounts and ARB/HPC ratios for ciprofloxacin, azithromycin, tetracycline, penicillin, and cephalosporin resistance in resident water illustrated that the prevalence of ARB occurred in pipe network water during stagnation. As reported by Zhang et al., the counts of ARB in the inlet water and outlet water of tap water in a simulated chlorinated system were 8.7 × 10^1^ to 4.15 × 10^3^ CFU/mL and 2.28 × 10^2^ to 7.07 × 10^3^ CFU/mL, respectively [33]. Xi et al. reported that of the HPCs tested, 0.04%–3.78% were resistant to tetracycline, which was lower than that for ciprofloxacin (0.18%–13.14%), amoxicillin (3.02%–15.22%), chloramphenicol (0.75%–13.96%), gentamicin (2.18%–13.40%), rifampin (14.23%–82.15%) and sulfisoxazole (0.12%–7.85%) [6]. 

As shown in Figure 3, long-term stagnation (48 h) of bacteria in water samples led to higher ARB counts and ARB/HPC ratios to the five antibiotics. However, different STs affected ARB distribution to the five antibiotics (Figure 3A). For W_ST-12h_, the ARB/HPC ratios of three types (ciprofloxacin, azithromycin and tetracycline) increased insignificantly (P > 0.05) compared with that for W_0_, which demonstrated that no significant increase in these ARB/HPC ratios occurred during water stagnation for 12 h at nighttime. For penicillin and cephalosporin, their ARB/HPC ratios in W_0_ were 13.1% and 6.4 %, respectively, and decreased to lower values in W_ST-12h_ (8.1% and 4.5%, respectively) (P > 0.05). Meanwhile, as the ST increased to 48 h, the ARB/HPC ratios of penicillin and cephalosporin in resident water increased to 49.4% and 58.6%, respectively, which were significantly increased compared with the value in W_0_ (P < 0.01). The reason for the lower bacterial resistance to penicillin and cephalosporin in water samples may be related to their hydrolysis by the extended-spectrum β-lactamases (ESBLs) [34]. For penicillin, its main antibacterial spectrum is Gram-positive bacteria, and it has little effect on water-based Gram-negative bacteria, but *Proteobacteria* has the highest proportion as Gram-negative bacteria in this study. According to reports, due to a similar structure of penicillin and cephalexin to β-lactams, they can be enzymatically hydrolyzed by the β-lactamase enzyme similar to acid hydrolysis in water [35]. Furthermore, β-lactamase enzyme dissemination on plasmids across a range of Gram-negative pathogens [36].

In addition, the ARB/HPC ratio for tetracycline in W_0_ and W_ST-48h_ was only 3.45% and 24.85%, respectively, which were significantly lower (P < 0.05) than the ratios of the other four antibiotics. Reinthaler et al. found that resistance to tetracycline was higher than all the antimicrobial substances in *Escherichia coli* isolated from three wastewater treatment plants in Australia, with the highest rate of resistance being 57% [37]. Lu et al. reported that survival of heterotrophic bacteria to 16mg/L tetracycline was almost 0 in the secondary effluents of two sewage treatment plants in Beijing, which was lower than those of chloramphenicol, penicillin, cephalosporin and ampicillin [38]. Xi et al reported that the HPC resistant to tetracycline was between 0.04% and 3.78%, which was lower than that of other antibiotics such as ciprofloxacin, amoxicillin, chloramphenicol, gentamicin, rifampin and sulfisoxazole [6]. These results agree with our findings for tetracycline. Although tetracycline has been used since 1940, the ARB/HPC ratio of tetracycline was not as high in previous research as it was in our study, which may be related to many factors, such as the dose and treatment course of tetracycline in the water source, and it was confirmed by research that the large amount of tetracycline resistance genes could be removed in drinking water treatment plants compared with other kinds of resistance genes [39]. In addition, as reported by Zhang et al., UV degradation of tetracycline resistance genes was significantly higher than that of other ARGs, which was mainly related to the number of pyrimidines in resistance genes [12]. These factors may have contributed to the low concentration of tetracycline ARB in fresh water and resident water in our study.

#### 3.3.2. Antibiotic Resistance Level

**Figure 4:**
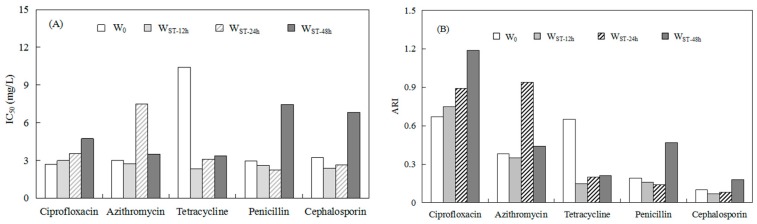
Antibiotic resistance level in water samples with different STs. (**A**) IC_50_ Counts. (**B**) ARI.

As shown in Figure 4A, the IC_50_ range for the five types of antibiotics in W_0_ was 2.7–10.4 mg/L, while the IC_50_ range in W_ST-12h_, W_ST-24h_, and W_ST-48h_ was 2.4–3.0 mg/L, 2.2–7.5 mg/L and 3.4–7.5 mg/L, respectively, higher than the antibiotic concentration range detected in fresh and residential water in previous studies. At present, the concentration of antibiotics detected in pipe network water reported by several countries ranges from 0.16 to 679.70 ng/L [40,41,42]. In addition, it was reported that the concentrations of tetracyclines and sulfonamides were 0.44–2.69 μg/L and 0.97–1.96 μg/L, respectively, in Shanghai’s Huangpu River, China [43]. Hence, the ARB can exist in fresh water and resident water for a long time with low concentrations of antibiotics in pipeline water. Furthermore, the low concentration of residual antibiotics in water can continue to exert selective pressure, and biofilms in the pipeline can also increase bacterial antibiotic resistance and transmission by harboring ARB, or promoting horizontal gene transfer of ARGs among bacteria, which causes and accelerates the appearance of ARB and ARGs abundance [33,44]. 

The ARI reflects the composite resistance level of HPC, while a higher ARI indicates greater resistance of HPC to a certain antibiotic. As shown in Figure 4B, the ARI of W_0_ for five types of antibiotics was in the range of 0.1–0.7, and that of W_ST-12h_, W_ST-24h_, and W_ST-48h_ was 0.07–0.8, 0.08–0.9 and 0.2–1.2, respectively. Furthermore, different STs affected the ARI of the different antibiotics tested (Figure 4B). When the ST was 12 h, the ARI of tetracycline, penicillin, cephalosporin and azithromycin, but not ciprofloxacin , in overnight water was low. This demonstrated that the HPC in the water samples after stagnation for one night were more sensitive to antibiotics and less tolerant. This was related to the change in water environment from fresh water to resident water during the 12 h stagnation period at nighttime, and bacterial stress contributed to reducing both ARB growth and resistance. However, with increasing the ST to 24 h, resistance increased, and azithromycin reached 0.94, exceeding the values observed at all other STs. With regard to the ST of 48 h, the increase in antibiotic resistance to ciprofloxacin, penicillin, and cephalosporin due to long-term stagnation seems to be related to biofilm having fallen off in the pipeline. The enhanced antibiotic resistance of biofilm bacteria, relative to floating (planktonic) bacteria, results in more health risks due to the protection of the biofilm from disinfection. As reported, when bacteria form biofilms, their resistance to most antibiotics can be greatly increased [45]. In addition, with increased ST, the ARI of ciprofloxacin was gradually enhanced, and a high value ARI (near or above 1) for ciprofloxacin indicated that the resistance of HPC to ciprofloxacin in W_ST-48h_ was high when compared with the standard recommended by CLSI. For tetracycline, the highest ARI was detected in W_0_, which then decreased with increasing ST. This is in agreement with the variations shown in Figure 3, where ARB counts and ARB/HPC ratios for tetracycline resistance were lower than other tested antibiotics at the same stage. These results show that the bacteria in resident water had low resistance to tetracycline during the ST of 12–48 h. As the ST continued to increase, the ARI for penicillin and cephalosporin in water samples increased, and their highest ARI was detected in W_ST-48h_, which was also due to the structure of the two antibiotics which are easily hydrolyzed and the exchange of fresh water during daytime stagnation, which may have increased the concentrations of antibiotics again. 

### 3.4. Redundancy Analysis (RDA) of Bacterial Communities and Antibiotic Resistance

**Figure 5:**
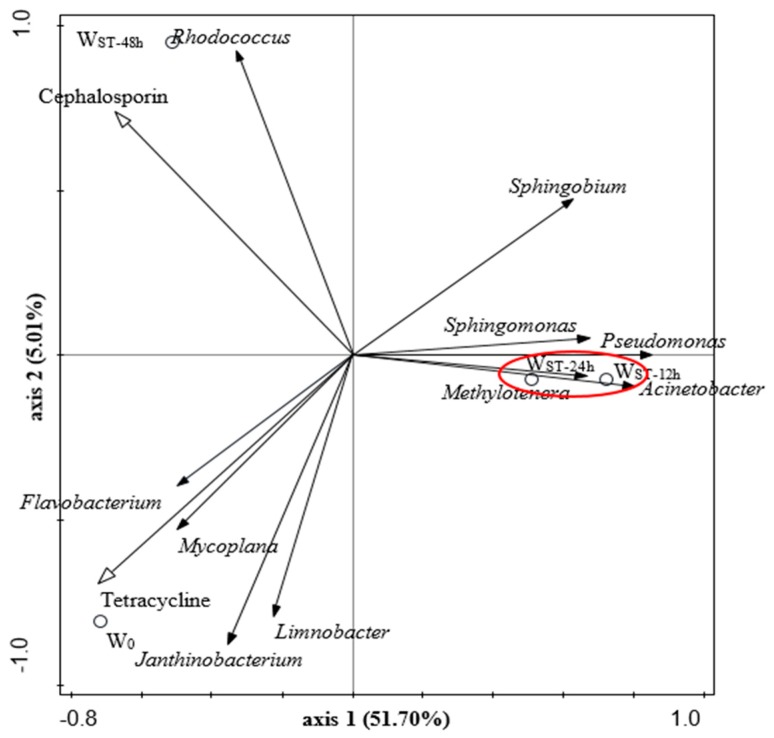
RDA ordination diagram of bacterial communities associated with environmental variables.

According to the RDA analysis of microbial community structure and ARI, the main microflora can be divided into three clusters (I, II, III) (Figure 5). In this study, two environmental variables with statistical significance, the ARI of tetracycline and cephalosporin, were selected for RDA analysis. It can be seen from Figure 5 that the distance between the two short-term samples of W_RT-12h_ and W_ST-24h_ was close, indicating that the microbial community structure was not affected by the ST of 12 and 24 h. This was similar to the two short-term samples in the analysis shown in Section 3.2. The distance between the samples of fresh water W_0_ and long-term resident water W_ST-12h,_ W_ST-24h_ was great, which indicates that the ST of 12 and 24 h had a significant influence on the microbial community structure compared with W_0_. This is in agreement with the aforementioned analysis in Section 3.2. In the RDA analysis plot, the correlation of microbes and ARI variables was represented by the cosine of the angle between their arrows. As shown in Figure 5, *Rhodococcus* in cluster I had a positive correlation with the ARI of cephalosporin, but a weak negative correlation with the ARI of tetracycline. Except for *Flavobacterium*, *Mycoplana* and *Sphingobium*, the genera in clusters II and III showed a weak negative correlation with the ARI of cephalosporin, while *Flavobacterium* and *Mycoplana* in cluster II showed a significant positive correlation with the ARI of tetracycline. This is in agreement with the high ARI of tetracycline and the high abundance of *Flavobacterium* and *Mycoplana* in W_0_. The genera in cluster III were negatively correlated with the ARI of tetracycline, and *Sphingobium* was negatively and significantly correlated with the ARI of tetracycline. In addition, the angle between the ARI of tetracycline and cephalosporin was about 90°, indicating that there was no correlation between them. Thus, this meant that few bacteria in fresh water and resident water were resistant to both tetracycline and cephalosporin. 

## 4. Conclusions

Response of the bacterial community and antibiotic resistance due to stagnant water in a municipal pipeline with different STs were investigated. Bacterial growth was observed during water stagnation at different STs. The composition of the bacteria community in overnight stagnant water under different STs was basically similar, and the predominant bacteria in fresh water and resident water were *Proteobacteria* and *Bacteroidetes*. However, during a ST of 12 h, bacteria related to fermentation were observed. When the ST increased to 48 h, the fermentation bacteria decreased, which was related to the exchange of pipe network water during daytime stagnation. Furthermore, enhanced ARB amounts and ARB/HPC ratios for five antibiotics in resident water were observed which illustrated that a prevalence of ARB occurred in the pipe network water during stagnation. ARI determination showed that the bacterial community had a high resistance level to tetracycline in fresh water, and then decreased during water stagnation. When the ST increased to 12 h, all ARI values of the five antibiotics were low, which was related to the change in parameters during water retention and reduced resistance during short-term stagnation. When ST was increased to 24 h, the ARI of azithromycin was highest, and when the ST increased to 48 h, the ARIs of ciprofloxacin, penicillin and cephalosporin were highest. Taken together, these findings show that bacterial growth and antibiotic resistance increased during water stagnation in a drinking water distribution network, which increased drinking water safety risks and is of significant concern.
